# Structural and Functional Characterization of the Redβ Recombinase from Bacteriophage λ

**DOI:** 10.1371/journal.pone.0078869

**Published:** 2013-11-11

**Authors:** Kazuko Matsubara, Ali D. Malay, Fiona A. Curtis, Gary J. Sharples, Jonathan G. Heddle

**Affiliations:** 1 Heddle Initiative Research Unit, RIKEN, Wako, Saitama, Japan; 2 School of Biological and Biomedical Sciences, Department of Chemistry, Durham University, Durham, United Kingdom; University of Massachusetts Medical School, United States of America

## Abstract

The Red system of bacteriophage λ is responsible for the genetic rearrangements that contribute to its rapid evolution and has been successfully harnessed as a research tool for genome manipulation. The key recombination component is Redβ, a ring-shaped protein that facilitates annealing of complementary DNA strands. Redβ shares functional similarities with the human Rad52 single-stranded DNA (ssDNA) annealing protein although their evolutionary relatedness is not well established. Alignment of Rad52 and Redβ sequences shows an overall low level of homology, with 15% identity in the N-terminal core domains as well as important similarities with the Rad52 homolog Sak from phage ul36. Key conserved residues were chosen for mutagenesis and their impact on oligomer formation, ssDNA binding and annealing was probed. Two conserved regions were identified as sites important for binding ssDNA; a surface basic cluster and an intersubunit hydrophobic patch, consistent with findings for Rad52. Surprisingly, mutation of Redβ residues in the basic cluster that in Rad52 are involved in ssDNA binding disrupted both oligomer formation and ssDNA binding. Mutations in the equivalent of the intersubunit hydrophobic patch in Rad52 did not affect Redβ oligomerization but did impair DNA binding and annealing. We also identified a single amino acid substitution which had little effect on oligomerization and DNA binding but which inhibited DNA annealing, indicating that these two functions of Redβ can be separated. Taken together, the results provide fresh insights into the structural basis for Redβ function and the important role of quaternary structure.

## Introduction

Homologous recombination (HR) mediates the pairing and exchange of DNA strands between homologous partners. HR is universally employed by organisms to preserve genomic information by restoration of DNA breaks, facilitation of recovery after interruptions to DNA replication and also as a means of generating genetic diversity [1], [2], [3].

Bacteriophage λ Red (recombination deficient) is a HR system consisting of three genes: *exo*, *bet* and *gam* encoding proteins Redα, Redβ and Redγ respectively. Redα is a 5′ to 3′ exonuclease which digests one of the two strands of the substrate DNA duplex, yielding ssDNA. Redβ is the recombinase in this process; a single stranded annealing protein (SSAP) with a subunit molecular weight of approximately 30 kDa which binds ssDNA and pairs it to complementary sequences [3]. Redγ protects the DNA ends by inhibiting the host RecBCD nuclease [4]. Redβ alone is sufficient to mediate recombinational exchange if suitable ssDNA is available [5].

HR by the Red system has been extensively reviewed [3], [6], [7] and can progress via either RecA-dependent (utilizing the bacterial strand exchange recombinase RecA for pairing) or RecA-independent pathways [8]. These follow strand invasion or single stranded annealing (SSA) routes, respectively, to recombinant formation (although *in vitro* experiments show that Redβ can carry out strand invasion even in the absence of RecA [9]). Annealing requires considerably less than 50 nt of sequence homology [5], [10], [11], [12].

The Red system is of interest for a number of reasons. Firstly, viral recombinases make an important contribution to evolution, both of the viruses and their hosts [13]. Secondly, they allow the addition or removal of DNA segments *in vivo*, a method that does not require the restriction digestion and ligation steps necessary when similar manipulations are carried out *in vitro* [14]. This approach is known as “recombineering” (recombination-mediated genetic engineering) [15] and is particularly useful for generating gene knockouts and in the manipulation of sizeable DNA molecules such as BACs [16]. Finally, Redβ is of interest because of its potential to shed light on general mechanisms of SSAPs.

The Red system has been the focus of considerable research and while Redα is well characterized [17], [18], [19] and its crystal structure in complex with substrate DNA has been solved [20], the mode of action of Redβ remains obscure. While it clearly performs the key annealing step in recombination, the mechanistic details are unclear, partly because only relatively low resolution structures from transmission electron microscopy (TEM) [21] and atomic force microscopy (AFM) [22] are available. These studies suggest that in the absence of ssDNA, Redβ forms a ring or split-lock washer-shaped multimer consisting of approximately 11 or 12 monomers. In the presence of ssDNA, the protein has been reported to become disordered [22] or form larger rings consisting of 15–18 subunits, depending on the length of DNA provided [21]. Redβ mixed with long complementary ssDNA molecules generates extended, left-handed helices thought to consist of protein and double stranded DNA (dsDNA) [21]. Proteins involved in strand pairing are typically toroidal or helical in shape, with a ring structure for phage recombinases first demonstrated for Erf from phage P22 [23]. A number of other ring-shaped recombinases are able to form extended nucleoprotein filaments in the presence of DNA and include Rad51, Rad52, RecA and Mgm101 [24], [25], [26], [27].

Redβ has been proposed as a distantly-related homologue of Rad52, a eukaryotic protein that promotes both single-strand annealing and stimulates homologous recombination reactions by assisting assembly of Rad51 on RPA-coated ssDNA [28], [29]. Like Redβ, Rad52 binds ssDNA and facilitates the annealing of homologous ssDNA [30]. Full-length human Rad52 (hRad52) consists of 418 residues, with a molecular weight of approximately 46 kDa. The crystal structures of the N-terminal 209 [31] and 212 [32] residues of hRad52 have been solved, revealing a toroidal arrangement composed of 11 subunits. The N-terminal region of Rad52 is evolutionarily conserved in eukaryotes [30] and is notable for having a positively-charged groove encircling the outer surface of the ring which most likely constitutes the ssDNA binding site [31], [32]. Rad52 also appears capable of associating with dsDNA via a nearby, yet distinct, binding site [33].

Among phage SSAP proteins, Sak from the virulent *Lactococcus lactis* phage ul36 has been classified as belonging to the same superfamily as Rad52 [34] and was subsequently identified as a genuine orthologue by further analysis [35], [36]. In contrast, despite similarities in activity, Redβ was assigned to a different SSAP superfamily [34]. However, studies by Erler *et al*. identified conserved elements in the N-terminal DNA binding domains of Redβ and Rad52 [22], while Lopes *et al*. also suggested that the Sak, Redβ and Erf families belong to the same Rad52 superfamily [37], although the alignments in the two reports differ, making it unclear if Redβ should indeed be considered as part of a larger superfamily that incorporates Rad52. An additional related protein, Mgm101, is also ring-shaped and is required for recombination of mitochondrial DNA [27].

In this present study, we have conducted the first detailed mutational analysis of the regions of Redβ predicted to share homology with Rad52 and have assessed their structure-function relationship. As a consequence we have identified key features of Redβ responsible for oligomerization, ssDNA binding and annealing that offer further insight into the mechanism of strand annealing by this superfamily of recombinases.

## Results

### Sequence Comparison between Rad52 and Redβ Proteins

**Figure 1:**
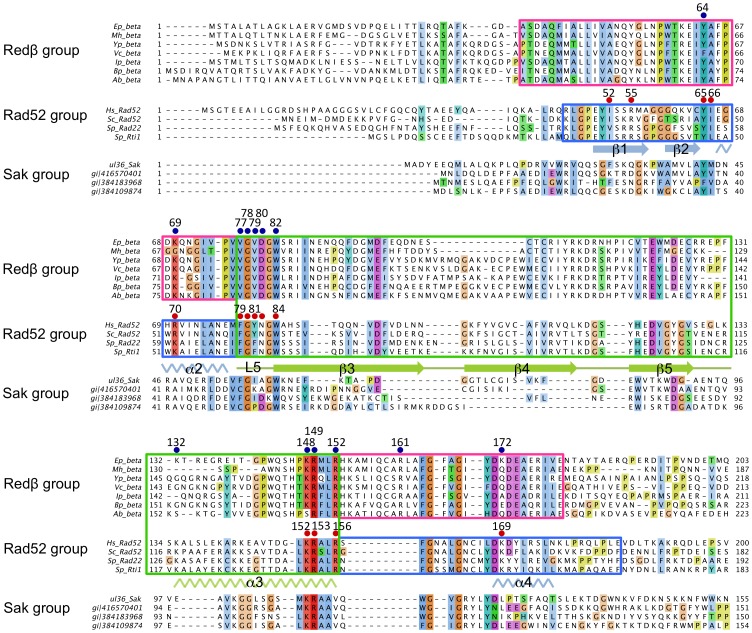
Comparison of Redβ, Rad52 and Sak N-terminal domains. Conserved residues are highlighted and colored according to the Clustal X scheme, with color intensity denoting the degree of homology between the three SSAP groups. The green boxed region indicates the stem structure of Rad52 [32]; secondary structure elements (β-β-β-α) in this region are highlighted. The blue boxed regions correspond to the conserved part of the domed cap region in the hRad52 N-terminal structure, while pink boxed regions indicate alternative conserved stretches in Redβ that flank the putative stem structure. Residues of Redβ mutated to alanine in this study are marked with blue circles and numbered accordingly. Residues of hRad52 that have been experimentally shown to be involved in ssDNA binding [32], [38] are indicated by red circles. Sak SSAP from phage ul36 and related sequences are also shown. Abbreviations and UniProt accession numbers: *Ep_beta*, Redβ from Enterobacteria phage λ (P03698); *Mn_beta*, bacteriophage recombinase from *Mannheimia haemolytica* (A7JWQ9); *Yp_beta*, DNA recombination protein from *Yersinia pestis* biovar Orientalis str. (A4IUY1); *Vc_beta*, putative DNA recombination protein from *Vibrio cholerae* (Q8KQW0); *Ip_beta*, phage recombination protein from Iodobacteriophage φPLPE (B5AX97); *Bp_beta*, putative phage recombination protein from *Burkholderia pseudomallei* strain 668 (A3NM00); *Ab_beta*, phage recombination protein Bet from *Acinetobacter baumannii*; *Hs_Rad52*, Rad52 homolog from *Homo sapiens* (P43351); *Sc_Rad52*, Rad52 from *Saccharomyces cerevisiae* (P06778); *Sp_Rad22*, Rad22 protein from *Schizosaccharomyces pombe* (P36592); *Sp_Rti1*, Rti1 protein from *S. pombe* (O42905); *ul36_Sak*, putative translation initiation factor from *Lactococcus* phage ul36 (Q9MC33_9CAUD). The three additional sequences in the Sak family are identified by GI accession numbers. The alignment display was generated using Jalview [48].

To help clarify the link between Redβ and Rad52, the degree of sequence conservation was reinvestigated as outlined in the Materials and Methods. Similar efforts to identify homologous features have been undertaken previously [22], [37]. The multiple sequence alignment presented in Figure 1 resembles most closely that generated by Erler *et al.* [22] and was used in subsequent analyses.

**Figure 2:**
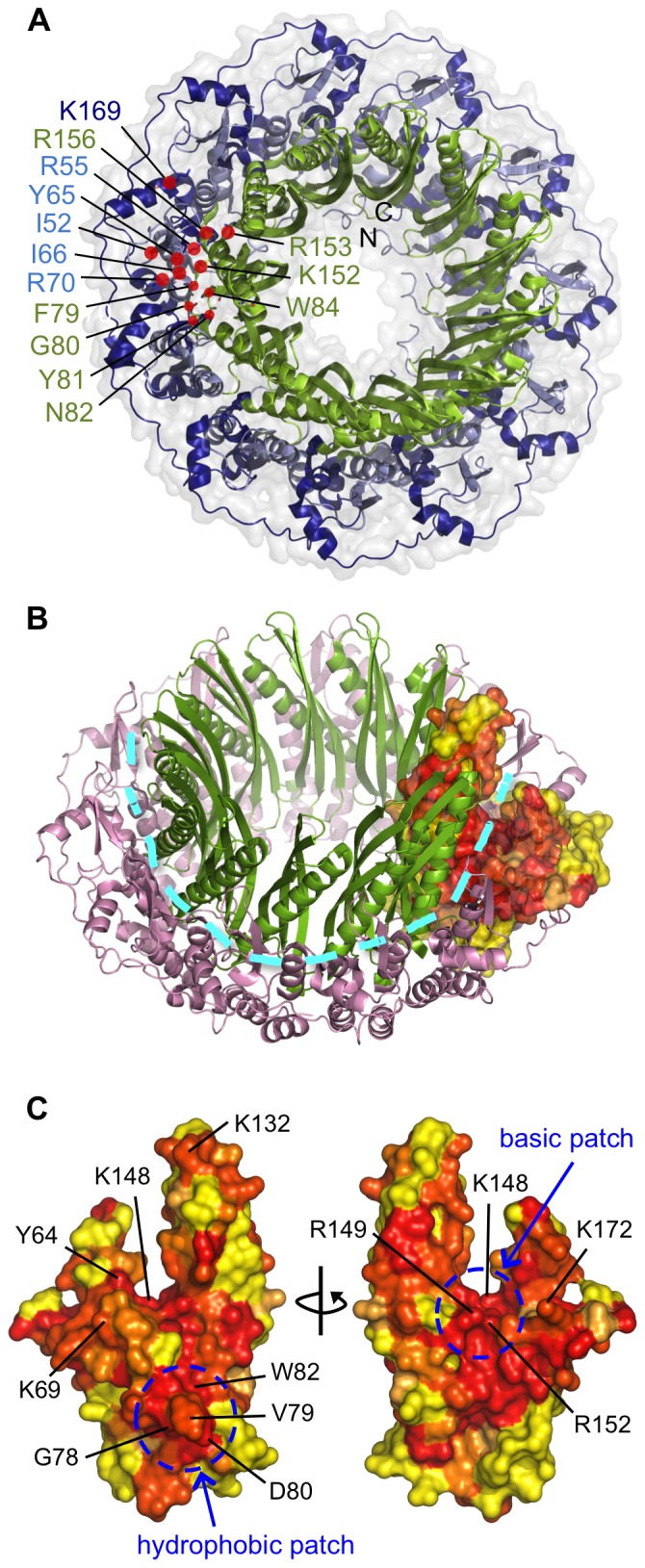
Comparison of hRad52 structure and homology model of Redβ. (A) Structure of undecameric N-terminal hRad52_1–212_ (PDB accession 1KN0) showing residues involved in ssDNA binding [32], [38]. The structure is colored as follows: residues 25–78 (part of the “domed cap”) in light blue, residues 79–156 (“stem structure”) in green, residues 157–220 (part of the “domed cap”) in dark blue. The N- and C- termini are also indicated. (B and C) Homology-based model showing sequence conservation between hRad52 and Redβ. (B) The hRad52_1–212_ oligomeric ring structure is shown, with the stem region in green, and the flanking regions in pink. One subunit is rendered in surface view and colored according to sequence conservation between Redβ and Rad52 family proteins based on the alignment in Figure 1 (yellow to red, least to highest degree of conservation), with the aid of the ConSurf server [49]. The basic groove predicted to bind ssDNA is indicated by a cyan dotted line. (C) Two views of a subunit of the 1KN0 structure (truncated to residues 25–177 for clarity) and colored according to the degree of conservation with respect to Redβ as in (B). The two conserved patches and predicted locations of the Redβ residues probed in this study are indicated. Residue V77 is buried and is therefore not displayed.

The N-terminal domain of eukaryotic Rad52 proteins is highly conserved and possesses ssDNA annealing activity [30], [31]. The X-ray crystal structures [31], [32] reveal an undecameric toroidal architecture resembling a mushroom, with a central ‘stem’ structure (residues 79–156) comprising the main body of the ring, and a ‘domed cap’ formed by the regions flanking the stem (residues 25–78 and 157–208; Figure 2A), as proposed by Kagawa *et al.* [32]. Based on the sequence alignment assembled here, it is apparent that the region with the greatest conservation between the two protein families corresponds to the stem structure of Rad52 (residues 77–152 in Redβ, boxed in green in Figure 1 and shown in green in Figure 2A). The sequence identity between Redβ and hRad52 is 15% over the entire N-terminal domain (residues 1–206 in Redβ) and 19.7% if only the stem regions are compared. Notably, the greatest similarity between the two sequence families is found at either end of this stem region. The first region maps to Redβ residues 76–82, with a predominantly hydrophobic consensus sequence of hhGhpGW (where h = hydrophobic, p = polar), corresponding to loop L5 in the Rad52 structure. The second region corresponds to Redβ residues 148–152, with a highly basic consensus sequence KRxLR (where x = any residue), and which includes residues known to be critical for ssDNA binding in Rad52 [32], [38]. Interestingly, the stem structure sequence is flanked by regions that are highly conserved within the respective Redβ and Rad52 families, but show significant divergence between the two families (blue and pink boxed regions in Figure 1). In Redβ, a highly conserved stretch of predominantly hydrophobic residues immediately precedes the putative stem region (Redβ residues 39–76). Immediately after the putative stem region there are an additional 10 conserved residues in Redβ (residues 154–163) that are not found in Rad52 (Figure 1). Crucially, residues that have been shown to be important for binding ssDNA in Rad52 [32], [38] and Sak [35] are also conserved in Redβ.

A number of conserved residues in Rad52, including I52, R55, Y65, I66, R70, F79, G80, Y81, N82, W84, K152, R153, R156 and K169, have been implicated in ssDNA binding [32], [38] (Figure 1 and 2A). Several of these residues (R55, Y65, K152, R153 and R156) line a positively charged groove encircling the hRad52_1–212_ structure and are proposed to contact the ssDNA substrate via backbone phosphates, leaving nucleotides exposed for strand annealing. In contrast, residues F79, G80, Y81, N82 and W84 form a hydrophobic cluster at the subunit interface in the undecameric hRad52_1–212_ ring that, in the crystal structure, is situated below the positively charged groove, making a direct interaction with DNA unlikely in that conformation. To verify the expected relationship between Redβ and Rad52 we performed alanine mutagenesis on residues which are predicted from the alignment to affect ssDNA binding: Y64, K69, V77, G78, V79, D80, W82, K148, R149 and R152 (Figure 1, 2B and 2C). Five of these (V77, G78, V79, D80 and W82) correspond to the previously mentioned hydrophobic cluster in the Rad52 structure, while the others lie in, or near, the positively-charged groove (Figure 2C). An additional substitution (R161A) was made based on findings indicating that this residue contributes to single-strand annealing *in vivo* [37], although it should be noted that while R161 is conserved within the Redβ family, it is absent from Rad52 proteins according to our sequence alignment (Figure 1). We also mutated residues K132 and K172, which could possibly correspond to hRad52 residues K133 and K169, respectively, that are implicated in dsDNA binding, ternary complex assembly, and D-loop formation [33], although sequence conservation between the two families in these regions is not clear (Figure 1).

### Purification of Redβ Proteins

Full-length Redβ wild-type (WT) protein and the 13 variants harboring alanine substitutions (Y64A, K69A, V77A, G78A, V79A, D80A, W82A, K132A, K148A, R149A, R152A, R161A and K172A) were expressed as N-terminal histidine-tagged proteins and purified by Ni^2+^-affinity chromatography, with subsequent removal of the tag by thrombin digestion as reported in the purification of Rad52 [25], leaving an additional three residues (Gly-Ser-His) prior to the initial methionine. Expression levels and solubility of all of the mutant proteins were similar to that of Redβ WT, with the exception of W82A, which showed poor expression and could not be purified in sufficient quantities for further study (data not shown). It is likely that W82 is critical for structural integrity, consistent with the orientation of the W84 side chain in the hRad52 structure.

The secondary structure of the purified Redβ proteins was probed using far-UV circular dichroism spectroscopy (CD). WT displayed the typical CD signature of a predominantly α-helical protein, as anticipated [39]. All of the mutants displayed a similar CD spectrum, suggesting no major alterations in protein folding (Figure S1).

### Effect of Mutations on ssDNA Binding

**Figure 3:**
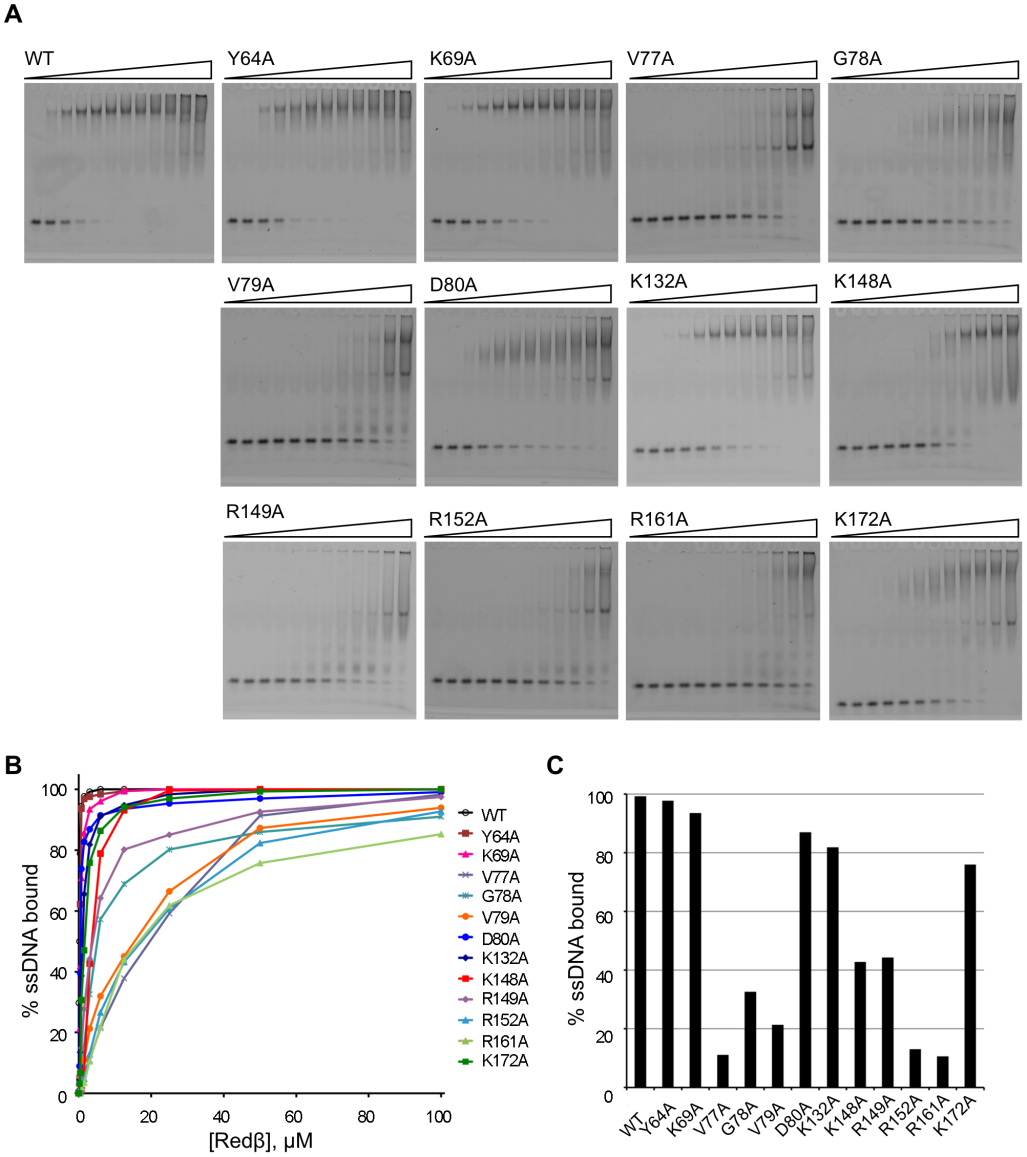
Binding of Redβ WT and mutant proteins to ssDNA. (A) Gel retardation assays showing binding of Redβ WT and mutant proteins to ssDNA. Varying concentrations of Redβ (0, 0.09, 0.19, 0.38, 0.75, 1.5, 3, 6, 12.5, 25, 50, 100 µM) were mixed with 10 nM 5′ Cy3-labeled 50 mer oligonucleotide as described in the Materials and Methods. Samples were fixed with 0.1% glutaraldehyde prior to separation on 6% PAGE. (B and C) DNA binding analysis based on quantification of the gels in (A). The percentage ssDNA bound by Redβ proteins at 3 µM is presented as a bar graph in (C).

The ssDNA binding activity of Redβ WT and the twelve stable mutant proteins was evaluated using electrophoretic mobility shift assays with a 50 nt ssDNA substrate (Figure 3). Samples were cross-linked with glutaraldehyde prior to electrophoresis, consistent with previous studies on Rad52 [32], [38]; in the absence of cross-linking only smears for the shifted species were observed (data not shown). Redβ WT formed a slow-migrating complex with ssDNA, with smearing indicative of some unstable protein-ssDNA interactions that disassociate during electrophoresis (Figure 3A). A similar complex, with some variation in stability, was observed with most of the mutants, although in some cases (e.g. V77A, R149, R152A) a discrete faster-migrating species was also evident. This latter complex can also be observed at high concentrations of the WT protein (Figure 3A). Y64A and K69A displayed only a minor reduction in ssDNA binding compared to WT (Figure 3), while the D80A, K132A, K148A, R149A and K172A mutants showed a more significant defect, binding 40–90% of the ssDNA at 3 µM protein (Figure 3C). The most severely impaired ssDNA binding variants were V77A, G78A, V79A, R152A and R161A, which bound only 10–35% of the ssDNA at the 3 µM data point (Figure 3C).

### Effect of Mutations on Binding a Second Strand of DNA

**Figure 4:**
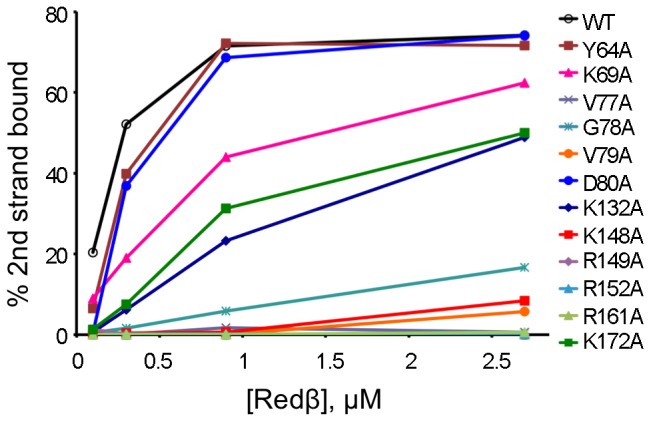
Binding of Redβ WT and mutant proteins to sequentially added complementary DNA strands. Gel retardation assays were performed using varying concentrations of Redβ (0.1, 0.3, 0.9, 2.7 µM) and 5 nM each of sequentially added complementary DNA strands. Images of the actual gels are shown in Figure S2.

The ability of the Redβ variants to form complexes with two 50 nt complementary oligos was investigated to probe the effects of the mutations on a second ssDNA binding site thought to be involved in strand annealing [40]. Redβ proteins were first incubated with Cy3-labeled ssDNA before addition of a second unlabeled strand (Figure 4, Figure S2). In contrast to the ssDNA binding assays, the complexes of Redβ with two complementary strands migrated as discrete high-MW bands during electrophoresis in the absence of cross-linking, reflecting the increase in stability of the complex when a second, complementary strand is present [40]. Overall, the pattern of binding was similar to that seen in the ssDNA assays (Figure 3). For instance, Y64A and D80A showing relatively modest decreases in binding in the presence of the second strand, whereas K69A, K172A and K132A showed greater decreases. The remaining mutants were significantly impaired in binding to the DNA, including K148A and R149A which show a much greater reduction in binding affinity compared with the results obtained with a single DNA strand (Figures 3 and 4).

### Effect of Mutations on DNA Annealing

**Figure 5:**
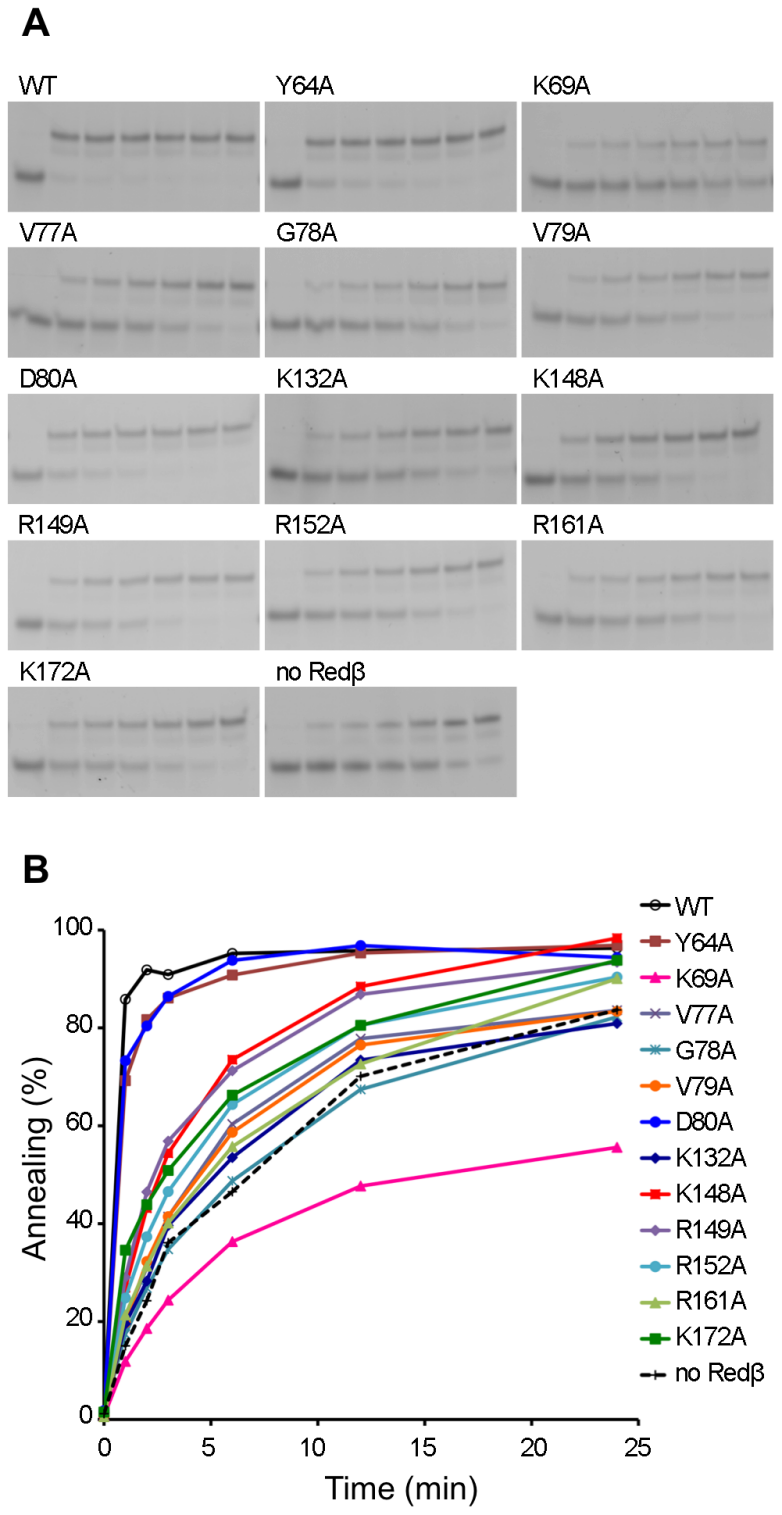
ssDNA annealing activities of Redβ WT and mutants. (A) Gel retardation assays showing formation of double-stranded DNA as a function of time. Lanes correspond to 0, 1, 2, 3, 6, 12, and 24 min time points. (B) Percentage of DNA annealing catalyzed by the Redβ variants as a function of time. Spontaneous annealing (without Redβ) is indicated by the dotted line.

In order to probe the effects of the mutations on ability to catalyze DNA annealing, assays were carried out with complementary 50 nt strands (Figure 5). The results revealed substantial annealing even in the absence of protein, with 50% of total DNA annealed at approximately 7 min. The presence of Redβ WT resulted in a significant acceleration in the rate, achieving 50% annealing after less than 1 min (Figure 5B). Mutants Y64A and D80A displayed activity similar to WT, consistent with the ssDNA binding assays (Figure 3B and 3C). Most of the other mutants showed 50% annealing at between 2–5 min, with K148A, R149A, and K172A having a moderate defect, whereas V77A, G78A, V79A and R161A exhibited a more severe impairment (Figure 5B), also consistent with the relative binding affinities for ssDNA. In contrast, K132A displayed a greater relative decrease in annealing activity compared to its ssDNA binding activity. The most striking result, however, was obtained with K69A. This mutant, while showing near-WT levels of ssDNA binding and a relatively moderate decrease in second strand binding ability (Figures 3 and 4), produced a significant inhibitory effect on DNA annealing (Figure 5B), taking approximately 15 min to reach 50%, considerably longer than the ssDNA in the absence of Redβ protein. Very little further increase in annealing was detected with K69A even when the reaction was incubated for a total of 96 min (data not shown).

### Subunit Structure of Mutant Proteins

**Figure 6:**
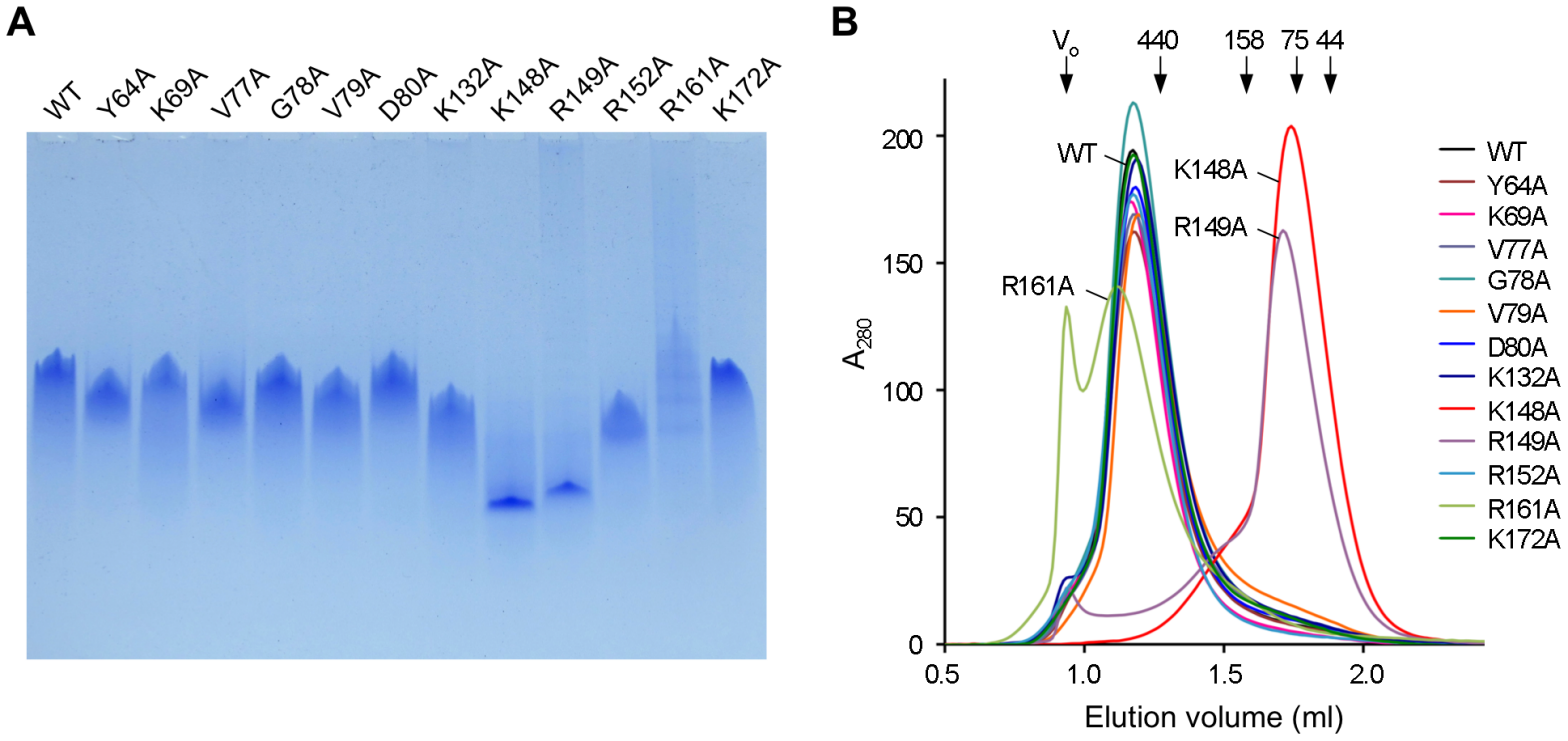
Characterization of the oligomeric structure of Redβ mutant proteins. (A) Native PAGE analysis of Redβ protein variants. Proteins (10 µg per lane) were separated on a 5–20% gradient polyacrylamide gel and visualized by staining with Coomassie Blue. (B) Size-exclusion chromatography of Redβ mutants. Proteins (10 µl of 6 mg/ml) were applied to a Superdex 200 5/150 GL gel filtration column in 20 mM potassium phosphate pH 6.0, 10 mM MgCl_2_, 0.15 M NaCl. Molecular masses were estimated by constructing a calibration curve from known molecular mass standards: ovalbumin (44 kDa), conalbumin (75 kDa), aldolase (158 kDa) and ferritin (440 kDa).

The oligomeric state of each of the variants was investigated since ring formation is likely to be important for proper functioning of Redβ. Analysis by native PAGE (Figure 6A) revealed that Redβ WT and the majority of the mutants (Y64A, K69A, V77A, G78A, V79A, D80A, K132A, R152A, and K172A) migrate as a single diffuse band, although there was some variation in the relative mobility of migrated species. In contrast, the majority of K148A and R149A appear as distinct bands of much lower molecular mass, with the remainder of the protein forming a ladder-like pattern of successively higher-order oligomers when larger amounts of protein are loaded (Figure S3). In addition, R161A showed a similar ladder pattern, but without a single predominant species (Figure 6A).

Size-exclusion chromatography has commonly been used to estimate the molecular weights of toroidal SSAPs [38], [41], [42], [43] and was employed here to further probe the quaternary structure of the Redβ variants (Figure 6B). Consistent with the native PAGE results, Redβ WT and most of the mutants (Y64A, K69A, V77A, G78A, V79A, D80A, K132A, R152A and K172A) eluted as a single peak of approximately 600 kDa. In the case of full-length hRAD52, similarly large-sized peaks have been attributed to complexes of several heptameric rings [38]. Redβ is known to form 11 or 12 mer rings in the absence of DNA [21], [22], potentially indicating that the 600 kDa peak is comprised of two or more 11 or 12 mer rings. (Figure S4). Interestingly, a two-ring complex has been proposed for the phage ul36 Sak protein [36]. Consistent with the native PAGE results (Figure 6A), K148A and R149A mutant proteins yielded single peaks of considerably lower molecular mass than WT, corresponding to complexes of approximately 3 subunits (Figure 6B). Also as seen with native PAGE, R161A exhibited an altered oligomeric state, eluting as two major peaks, one at the void volume corresponding to aggregated material and the other with a molecular mass higher than WT (likely over 800 kDa). Taken together, these results suggest that Redβ WT in the *apo* form adopts a multimeric subunit structure, which is disrupted by mutation of either K148 or R149. In addition, the R161A mutant, despite folding normally (Figure S1), is prone to aggregation, consistent with its poor DNA binding and annealing activity. The remaining Redβ mutants do not appear to significantly affect multimer formation.

### Transmission Electron microscopy (TEM) Analysis

**Figure 7:**
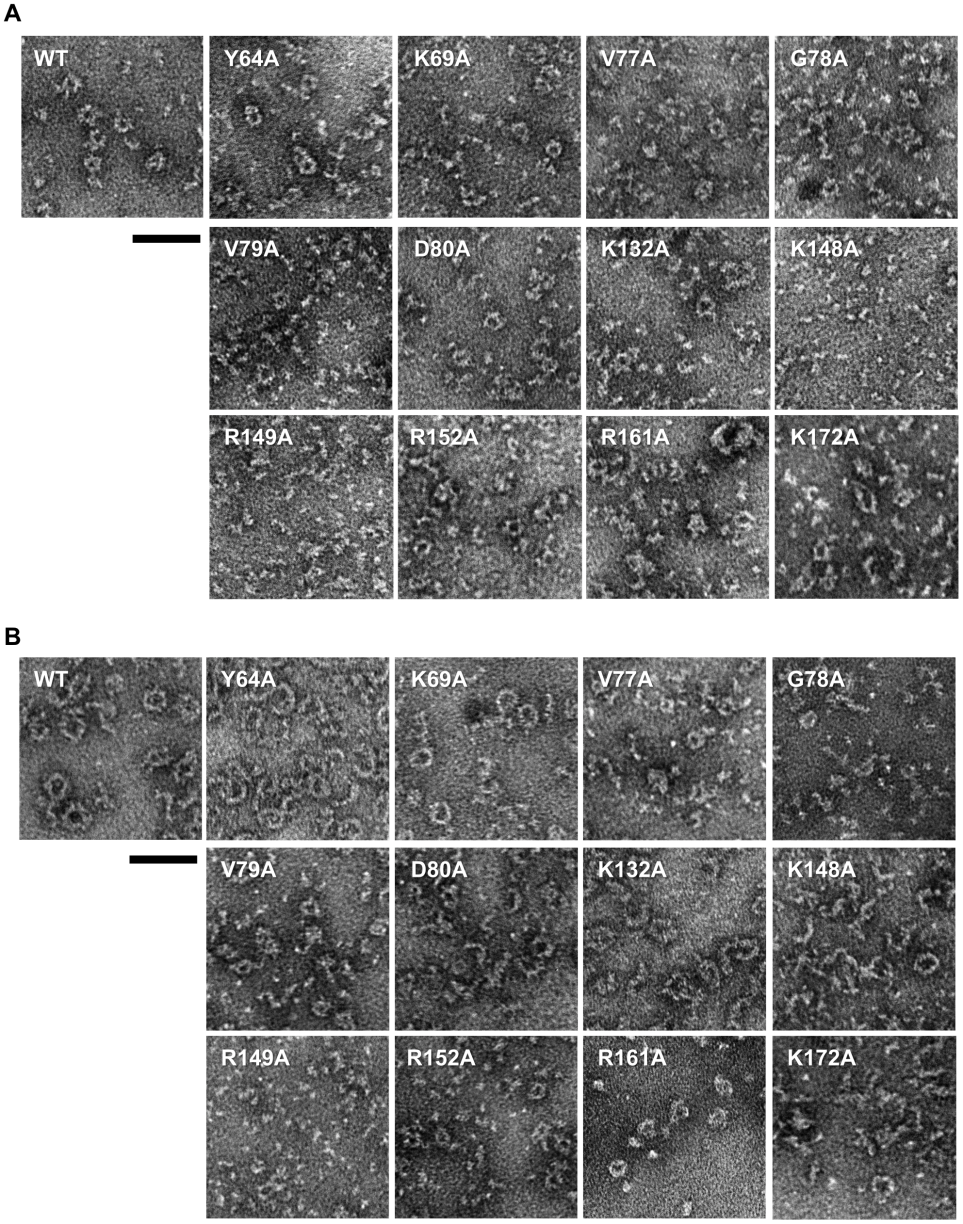
Visualization of Redβ WT and mutant protein oligomers by TEM. Images were obtained for proteins (2 µM) alone (A) or in the presence of 1 µM 50 nt ssDNA (B). The scale bars represent 50 nm.

We used TEM for direct visualization of the quaternary arrangement of Redβ variants in the presence or absence of the 50-nt ssDNA substrate (Figure 7). The *apo* WT protein was observed as a heterogeneous mixture of structures that includes numerous toroids with an average outer diameter of 12 nm (range = 7.7–15.6 nm) similar to the dimensions reported previously [21] (Figure 7A). The majority of the mutants, including K69A which exhibited an inhibitory effect on annealing, displayed similar ring structures, although some mutants showed a greater number of incomplete or non-ring structures. In the case of R161A, WT-like rings were observed alongside larger protein aggregates, while for the K148A and R149A mutants very few multimeric structures were observed, consistent with the results from both native PAGE and size exclusion chromatography.

Redβ WT complexed with ssDNA oligonucleotides has been variously reported to form ring structures of 15–18 subunits [21] or disordered structures [22]. In our TEM images, WT protein mixed with 50 nt ssDNA produced distinct rings and filaments that presumably constitute nucleoprotein complexes (Figure 7B). These structures differ from those seen in the *apo* form, the filaments being thicker (2.0–2.5 nm) and the rings having a larger average outer diameter of 16 nm. In the case of the mutant proteins the propensity to form filaments and larger rings in the presence of ssDNA varied considerably. Several displayed a similar appearance to WT (Y64A, K69A), while others formed mainly incomplete rings or filaments (D80A, K132A, K148A, K172A). In contrast, some mutants appeared largely unchanged from their *apo* structures, suggesting limited nucleoprotein complex formation (V77A, G78A, V79A, R149A, R152A, R161A). Overall, the TEM data correlate well with the ability of the substitution mutants to interact with ssDNA in the electrophoretic ssDNA binding experiments. One notable finding is that K148A is able to form a complex with ssDNA despite having a disrupted quaternary structure in the *apo* form.

## Discussion

Although the question of how SSAPs, and in particular Redβ, function to generate recombinants using relatively short regions of nucleotide sequence homology is fundamentally important, a detailed structure-function analysis, beyond that of functional domains [44], has so far been lacking. One approach has been to clarify the evolutionary relationships between Redβ and other SSAPs, although this has led to somewhat conflicting results.

Our own analysis suggests key similarities between the N-terminal domain of Rad52 and those of the phage SSAPs Sak and Redβ. We propose that Rad52, Sak and Redβ represent three distinct but related families within a larger superfamily, which although exhibiting limited sequence homology, share a conserved architectural core and a similar mode of action.

To test our hypotheses, 13 residues in Redβ were selected as targets for mutagenesis and the purified mutant proteins assessed experimentally for their impact on DNA binding and annealing. The results of these investigations, summarized in Table S1, largely validate the functional importance of the conserved residues identified in the sequence alignment. However, several notable and somewhat counterintuitive results were obtained whose implications will be discussed below.

For the hydrophobic cluster (Redβ residues 77–82), the corresponding residues in hRad52 (79–84) are localized at the interface between two adjacent subunits in the toroidal structure; in particular the large side-chain of Y81 (V79 in Redβ) inserts into a hydrophobic cavity of an adjacent subunit, in a ball-and-socket manner likely to be important in maintaining the oligomeric ring configuration. Despite this, our results reveal that the corresponding point mutants in Redβ retain an intact oligomeric structure yet display a loss of DNA binding ability, which is particularly severe with residues V77, G78 and V79 but only mild in the case of D80. It should be noted that a similar phenomenon has been reported for the corresponding residues in full-length hRad52 [38]. In the related yeast mitochondrial DNA recombination protein Mgm101, mutation of the equivalent residue to Redβ V77 (F153A) likewise resulted in poor ssDNA binding [27], although this Mgm101 mutant also displayed aggregation which could have contributed to the defect. A possible explanation for the effect on DNA binding is that mutations at the hydrophobic region may lead to structural changes that indirectly affect the configuration of the ssDNA binding residues at the protein surface, making them less able to bind DNA. A second and related possibility is that the mutations may inhibit conformational changes that Redβ must make upon binding to ssDNA [21], [22]. The loss of annealing activity in mutants V77A, G78A and V79A most likely arises from their inability to bind ssDNA substrates. With respect to the lack of effect on oligomeric structure, it is possible that the C-terminal domains in full-length Redβ and Rad52 provide additional interactions that stabilize the quaternary structure despite mutations at the conserved hydrophobic subunit interface.

The conserved basic cluster in the Rad52 N-terminal structure, corresponding to Redβ residues 148–152, constitutes a groove encircling the oligomeric ring and represents the probable ssDNA binding site. Mutations at these residues in Redβ conferred a significant loss in DNA binding ability, consistent with the results obtained with Rad52 [32], [38], particularly for R152A. However, it should be noted that DNA annealing activity is not entirely abolished in these mutants. Both K148A and R149A exhibited an inability to form higher-order oligomeric species and showed defects in ring formation under TEM in the absence of ssDNA. In the case of R149A, the corresponding residue in the hRad52 structure (R153) participates in hydrogen bond interactions with a neighboring subunit, which might account for the defects in oligomerization [32]. However, this is not the case with K148A where the equivalent residue in hRad52 (K152) points towards the groove and does not appear to participate directly in intersubunit interactions. Indirect effects cannot be ruled out, especially given the proximity of the adjacent residue in each case, nor possible differences between the structures of Redβ and the N-terminal domain and full-length Rad52 proteins. Interestingly, the oligomeric status of the relevant mutants in Rad52 or Sak has not been reported. It should also be highlighted that the Redβ mutant K148A, despite the disruption of the native ring structure and a reduced DNA binding ability, showed the formation of nucleoprotein filaments in the presence of ssDNA under TEM. These results suggest that, at least in the case of Redβ, ssDNA binding and annealing may occur even without the capacity to assemble a complete toroidal structure. However, we cannot exclude the possibility that the inclusion of complementary ssDNA strands in a reaction has some stabilizing effect that promotes partial or complete ring formation to occur even though higher order structures were not observed under TEM in the presence or absence of a single DNA strand.

For residues outside the stem region, Redβ residue K69 merits special attention. Although the residue is conserved within the Redβ family of sequences, its correspondence to residue R70 of hRad52 is not entirely certain. From our results, Redβ K69A displays a WT-like oligomeric structure and shows only a moderate reduction in DNA binding, consistent with previous findings on Redβ [44]. However, in ssDNA annealing assays K69A displayed a marked inhibitory effect, such that rate of strand annealing was considerably reduced compared to the rate of spontaneous DNA annealing in the absence of protein. We propose that Redβ K69A retains the capacity to interact with two ssDNA molecules yet binds in such a way as to hinder annealing of the two strands. How this inhibition occurs and its implications for the mechanism of annealing by Redβ warrants further investigation.

Mutation at R161 (R161C) in Redβ has previously been reported to cause a dramatic decrease in single-strand annealing *in vivo* [37], although there is no obvious equivalent of this residue in hRad52 according to our analysis. Consistent with the published experimental data, we found a significant decrease in the DNA binding and annealing activities of an R161A mutant *in vitro* (Figures 3–5). Native PAGE, gel filtration chromatography and TEM (Figure 6 and 7) suggest that this mutation leads to the formation of abnormally large protein aggregates, although TEM results show that oligomeric ring formation is not itself impaired. From these results we deduce that the loss of DNA binding in R161A is a consequence of a large proportion of the protein being sequestered in non-functional aggregates.

From our results, the Redβ Y64A mutant only exhibited mild perturbations in DNA binding and annealing. In contrast, the corresponding mutation in hRad52, Y65A results in a drastic reduction in ssDNA binding activity [32], [38]. It is possible that, although tyrosines at this position are ostensibly conserved, they play different roles in the two protein families.

Similar overall effects were observed for the mutants K132A and K172A, which are only weakly conserved in our sequence alignments. In both cases, the mutation caused only a moderate reduction in ssDNA binding activity and relatively more significant effects on second strand DNA binding and annealing activity. Although poorly conserved, it is possible that these and other residues participate in a network of positively-charged residues on the surface of the protein that stabilize second-strand DNA interactions and thereby promote annealing. A previous study reported that K172A mutation leads to the abrogation of DNA binding in a sequential, two-strand DNA-binding assay with Redβ [44]. However, it should be noted that the mutant generated therein was made in a truncated Redβ background (residues 1–188) and removal of the C-terminal region may exacerbate the effects of the K172A mutation seen in our study.

Our gel filtration data suggest that Redβ WT exists predominantly as multimers of an 11–12mer ring, although a precise estimate of molecular mass by this approach is not possible. It is interesting to note that for Sak protein, both full-length and N-terminal versions are predicted to form mixtures of 11 and 22mers [36]. The fact that the crystal structure of the N-terminal part of Rad52 is an undecamer is important as the structural arrangement of the full-length (heptamer-forming) protein may differ significantly, with concomitant effects on the function of specific regions and residues. The results in this study may reflect a conformation of Redβ in terms of intra-and inter-subunit interactions that most closely resembles that of full-length Rad52 although with 11–12-fold, rather than 7-fold, rotational symmetry.

Existing HR models suggest that a single SSAP ring binds to one ssDNA strand [31] before a second strand is positioned and anneals. Models for the annealing mechanism of these proteins often invoke two rings somehow acting in tandem with one ring binding to each of the single strands to be annealed [45], [46]. Further studies with appropriate DNA substrates will be required to understand if this is the case in Redβ.

The work presented here clarifies the relationship between Redβ, Sak and Rad52 SSAPs and offers the first study to validate this relationship experimentally by alanine mutagenesis of Redβ. Residues in Redβ conserved between the three protein families show similar functionality, although several novel features are apparent. Significantly, residues at the subunit interface are linked to DNA binding while others implicated in DNA binding in Sak and Rad52 were also associated with oligomer formation. These results provide the basis for further work to clarify the oligomeric states of Redβ and related proteins including a better understanding of the structure of the full-length protein and the mechanism of ssDNA annealing.

## Materials and Methods

### Sequence Alignments

The sequences for full-length Redβ and hRad52 were searched separately against the RefSeq database in NCBI using PSI-BLAST; and the related sequences used to build the consensus alignment for each family were selected to obtain as broad a range of homologues as possible and at the same time avoid sequences that differed by only a few residues. Redβ family sequences were selected within a BLAST maximum score of 157–259 (maximum identity of 46–65%). The selected sequences were then aligned using the T-Coffee program [47]. For the Rad52 family, the criteria applied were a BLAST maximum score of 152–523 (maximum identity of 48–68%). In both cases conserved features within each family were noted. Subsequently, the two alignments were merged in Jalview [48] and the N-terminal domains were aligned using the Clustal module, in addition to manual alignment using the conserved residues as a guide.

### Site-directed Mutagenesis of the *Bet* Gene

The *bet* gene was amplified from phage λ genomic DNA using Pfx DNA polymerase and oligonucleotides 5′-TAAAACATATGAGTACTGCACTCGC-3′ and 5′-TGCAGGATCCTGTCCGGTGTCATGC-3′. The PCR product was digested with *Nde*I and *Bam*HI (underlined) and inserted into pGADT7 to create pFC142. The integrity of the cloned gene was confirmed by DNA sequencing. The insert from pFC142 was subcloned into the pET14b expression vector using the same restriction sites. The latter construct expresses the Redβ protein with an N-terminal 6-histidine tag. Alanine substitution mutants were generated using the Phusion high-fidelity PCR kit (Thermo Fisher Scientific). Each PCR contained 10 µg of the template (*bet* gene in pET14b), 0.26 µM each of the forward and reverse oligonucleotide primers containing the desired point mutation (Operon Biotechnologies), 200 µM dNTPs, 3% DMSO, 0.04 U/µl Phusion DNA polymerase and Phusion buffer in a total volume of 25 µl. The mixture was incubated at 98°C for 30 seconds followed by 18 cycles at 98°C for 10 seconds, 50°C for 30 seconds and 72°C for 3 minutes, followed by a final step at 72°C for 10 minutes. For some of the mutants an annealing temperature of 45 or 55°C was necessary. Ten units of *Dpn*I (New England Biolabs) was added to the completed PCR reactions which were incubated at 37°C for 1 hour. A portion of the digested sample was introduced into XL10-Gold Ultracompetent cells (Agilent Technology) and transformants selected on LB agar plates containing 0.1 mg/ml ampicillin. The successful introduction of each mutation was confirmed by DNA sequencing.

### Protein Purification

BL21(DE3) competent cells (New England Biolabs) were transformed with the WT polyhistidine-tagged *bet* construct and its mutant derivatives. Cells were pre-cultured overnight at 37°C in LB medium containing 0.1 mg/ml ampicillin, inoculated into 1 L of the same medium and cultivated to an A_600 nm_ of ∼0.7. Redβ protein expression was initiated by addition of isopropyl-β-D-thiogalactopyranoside to a final concentration of 0.4 mM and cells harvested by centrifugation after 3 hours. Cells were resuspended in histidine-tag binding buffer (20 mM Tris-HCl pH 8.0, 0.5 M NaCl, 30 mM imidazole) and lysed by sonication. The lysate was clarified by centrifugation and the supernatant applied to a HisTrap FF column (GE Healthcare). After washing in histidine-tag binding buffer, bound proteins were eluted with his-tag elution buffer (20 mM Tris-HCl pH 8.0, 0.5 M NaCl, 265 mM imidazole). Buffer exchange was performed using Amicon Ultra 10,000 MWCO spin columns (Merck Millipore) against 20 mM Tris-HCl pH 8.0, 0.5 M NaCl. The 17-residue histidine-tag moiety was removed by addition of 10 U thrombin (Nacalai Tesque) followed by incubation at 20°C for ∼16 hours. Uncleaved protein was removed by passage through a HisTrap FF column in histidine-tag binding buffer, while thrombin protease was eliminated subsequently using a Benzamidine FF column (GE Healthcare). Redβ protein samples were subjected to buffer exchange and concentration against 20 mM Tris-HCl pH 8.0, 1 mM DTT, 10% glycerol using Amicon Ultra 10,000 MWCO spin columns. A portion of the sample was kept at 4°C and used directly for CD analysis. The remainder of the purified protein samples were subjected to buffer exchange/concentration against 20 mM Tris-HCl pH 8.0, 1 mM DTT, 50% glycerol and stored at −20°C until further use.

### Circular Dichroism Spectroscopy

Purified Redβ proteins (0.1 mg/ml) in 20 mM potassium phosphate pH 6.0 buffer were placed in a quartz cuvette with a 0.1 cm path length and circular dichroism in the range of 500-190 nm was monitored at room temperature using a Jasco J-720 spectropolarimeter.

### Non-denaturing (native) PAGE

10 µg of protein was mixed with loading buffer (to a final concentration of 62.5 mM Tris-HCl pH 6.8, 10% glycerol, 0.002% bromphenol blue) and separated on a 5–20% gradient polyacrylamide gel (ATTO) using 25 mM Tris, 192 mM glycine as running buffer at 200 V, 30 mA for 1 h. The gel was stained with Coomassie Brilliant Blue R-250.

### Gel Filtration Chromatography

Redβ protein samples (10 µl of 4 mg/ml) were applied to a Superdex 200 5/150 column (GE Healthcare) in buffer containing 20 mM potassium phosphate pH 6.0, 10 mM MgCl_2_, 0.15 M NaCl. The molecular mass of eluting species was estimated from a standard curve constructed from a high molecular weight calibration kit (GE Healthcare) consisting of ferritin, aldolase, conalbumin and ovalbumin.

### ssDNA Binding Assay

Varying amounts of Redβ (including a control without protein) were mixed with 10 nM of a 50 nt single-stranded DNA substrate (5′-TGCGGATGGCTTAGAGCTTAATTGCTGAATCTGGTGCTGTAGCTCAACAT-3′) labeled with Cy3 at the 5′ end (Operon Biotechnologies). Binding mixtures (10 µl) were incubated for 40 minutes at 37°C in 50 mM potassium phosphate buffer pH 6.0, 5 mM EDTA, 5% glycerol, 0.1 mg/ml BSA. Samples were fixed with 0.1% glutaraldehyde and incubated for a further 20 min at 37°C. Loading buffer containing glycerol and xylene cyanol was added and samples separated on a 6% DNA retardation gel (Life Technologies) in 0.5×TBE buffer and visualized using a Molecular Imager FX (Bio-Rad). Band intensities were quantified using ImageJ (http://imagej.nih.gov/ij/); the area beneath a rectangle spanning both the bound and unbound substrate in each lane was calculated and used to determine the percentage DNA bound by Redβ protein. Similar results were obtained by quantifying only the amount of substrate in each lane.

### Second DNA Strand Binding Assay

Varying amounts of Redβ were mixed with 5 nM of 50 nt single-stranded DNA substrate labeled with Cy3 at the 5′ end (50-minus; the same substrate used for the ssDNA binding assay) in 20 mM Tris-HCl pH 7.5, 5 mM MgCl_2_, 1 mM DTT, 0.1 mg/ml BSA and incubated for 20 min at 37°C. Unlabeled 50 nt complementary DNA strand (50-plus) was added to 5 nM final concentration and further incubated for 40 min at 37°C. Samples (10 µl) were then electrophoresed and visualized according to the ssDNA binding assay protocol.

### DNA Annealing Assay

Reaction mixtures (70 µl total volume) containing 1.0 µM Redβ, 5 nM Cy3-labeled 50 mer oligonucleotide (50-minus; identical in sequence to that used in ssDNA binding assays), 20 mM Tris-HCl pH 7.5, 5 mM MgCl_2_, 1 mM DTT, and 0.1 mg/ml BSA were incubated at 37°C for 20 min. Unlabeled 50 nt complementary strand oligonucleotide (50-plus) was added at 5 nM final concentration, mixed and 10 µl aliquots of the reaction were removed at 1, 2, 3, 6, 12, and 24 min and stopped with 200 nM unlabeled 50-minus oligonucleotide, 0.3 U proteinase K (Nacalai Tesque) and 0.5% SDS. For zero time points the Cy3-labeled 50-minus oligonucleotide was premixed with excess unlabeled 50-minus oligonucleotide prior to addition of the other reaction components. A control reaction without Redβ was conducted in parallel. Samples were separated on native 10% PAGE in 1×TAE buffer at 200 V for 120 min and the bands visualized on a Molecular Imager FX (Bio-Rad). Intensities of the annealed and unannealed substrate bands were quantified using ImageJ and the percentage annealing calculated.

### TEM

Each Redβ mutant variant was dialyzed overnight against 20 mM potassium phosphate buffer pH 8.0 at 4°C. Samples for TEM observation were prepared with 10 µM protein incubated in the presence or absence of 5 µM of an unlabeled 50-minus ssDNA substrate described above and incubated at 37°C for 40 minutes in a 5 µl mixture containing 20 mM potassium phosphate buffer pH 6.0 and 20 mM MgCl_2_. The samples were then fixed by the addition of glutaraldehyde to 0.6% and incubated further at 37°C for 20 minutes. Reaction mixtures were diluted 5-fold in the same buffer and 4 µl applied to hydrophilized carbon-coated copper TEM grids (STEM Co.), blot-dried, negative-stained with 3% phosphotungstic acid (pH 6), blot-dried and allowed to air-dry. The samples were visualized using a JEOL JEM-1230 80 kV transmission electron microscope.

## Supporting Information

Figure S1 **Far-UV CD measurements of Redβ WT and mutant proteins.**
(TIF)

Figure S2 **Gel shift experiments for second DNA strand binding activity of Redβ WT and mutant proteins.** Electrophoresis was performed after the sequential addition of two complementary 50 nt DNA strands (5 nM each) to varying concentrations of Redβ (0.1, 0.3, 0.9, 2.7 µM).
(TIF)

Figure S3 **Native PAGE analysis using overloaded samples.** Redβ WT and mutant proteins (20 µg per lane) were loaded and run according to the method in Figure 6A. Ladder-like migration patterns were observed for mutants K148A, R149A, and R161A.
(TIF)

Figure S4 **Standard curve for estimation of molecular weights using gel filtration chromatography.** *K_av_* values [(*V_e_* - *V_0_*)/(*V_c_* - *V_0_*)] against log MW of the known protein standards. The generated linear equation and the R^2^ values are also indicated. The dotted line indicates extrapolation of the standard curve used to estimate the molecular weights of the larger eluting species, such as Redβ WT.
(TIF)

Table S1 **Summary of Redβ mutant analysis and the role of equivalent residues in hRad52.**
(DOCX)
