# Supplementary figures and images for: Structural and Functional Characterization of the Redβ Recombinase from Bacteriophage λ
Source: PLoS One. 2013 Nov 11;8(11):e78869. doi: 10.1371/journal.pone.0078869 (PMC3823998; doi:10.1371/journal.pone.0078869)

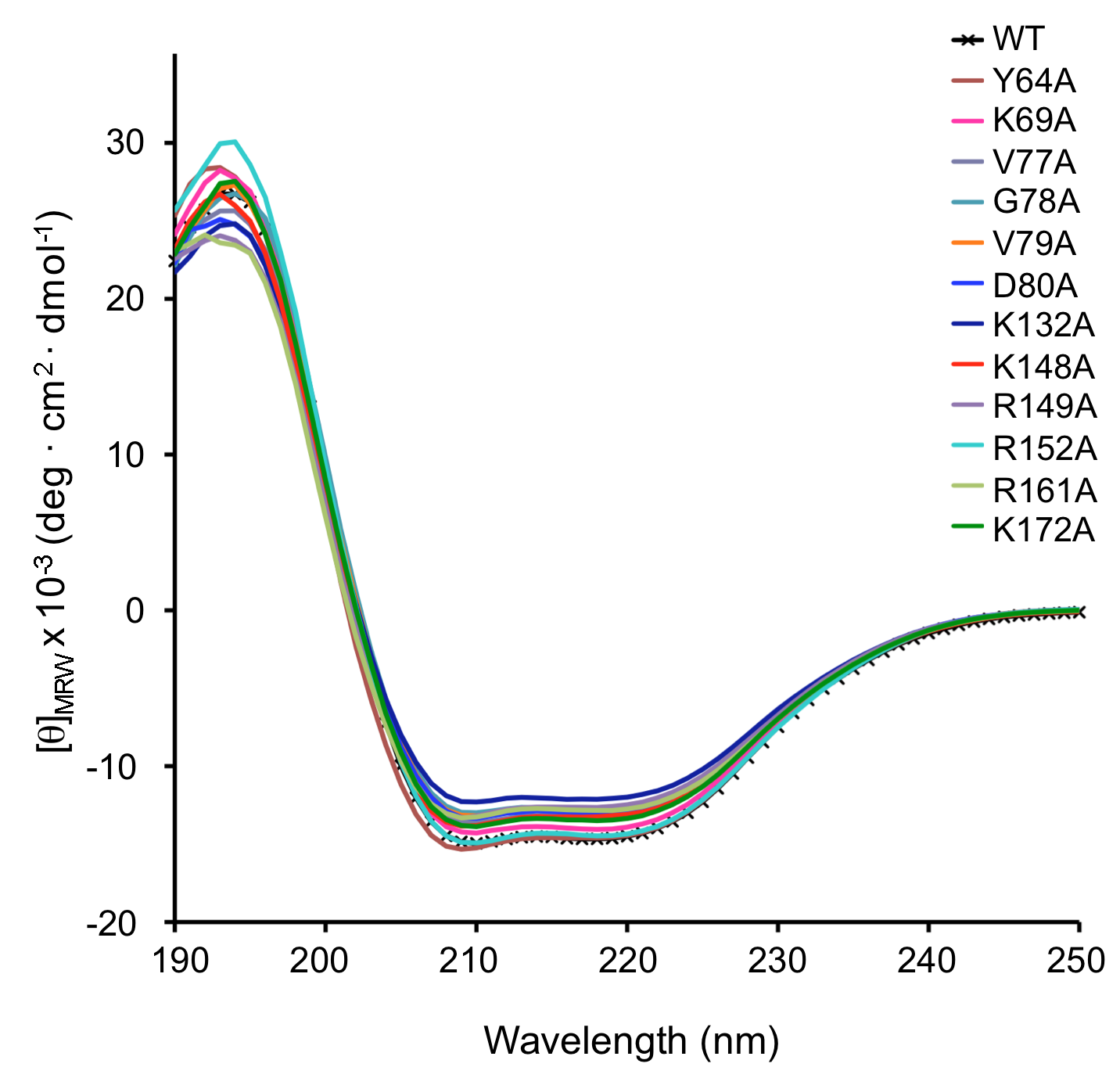

Supplement: Figure S1 — Far-UV CD measurements of Redβ WT and mutant proteins. (TIF) [file pone.0078869.s001.tif]

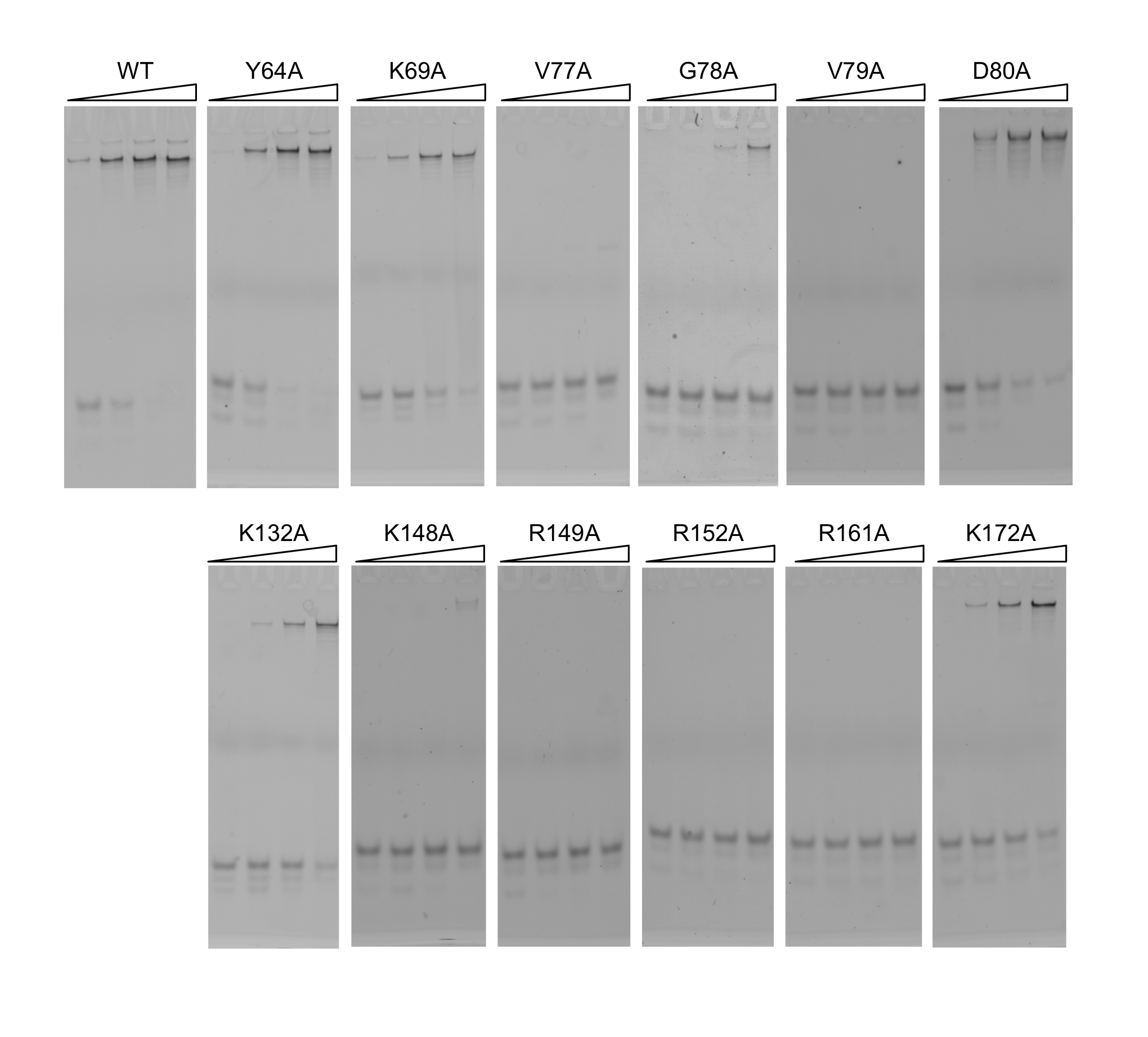

Supplement: Figure S2 — Gel shift experiments for second DNA strand binding activity of Redβ WT and mutant proteins. Electrophoresis was performed after the sequential addition of two complementary 50 nt DNA strands (5 nM each) to varying concentrations of Redβ (0.1, 0.3, 0.9, 2.7 µM). (TIF) [file pone.0078869.s002.tif]

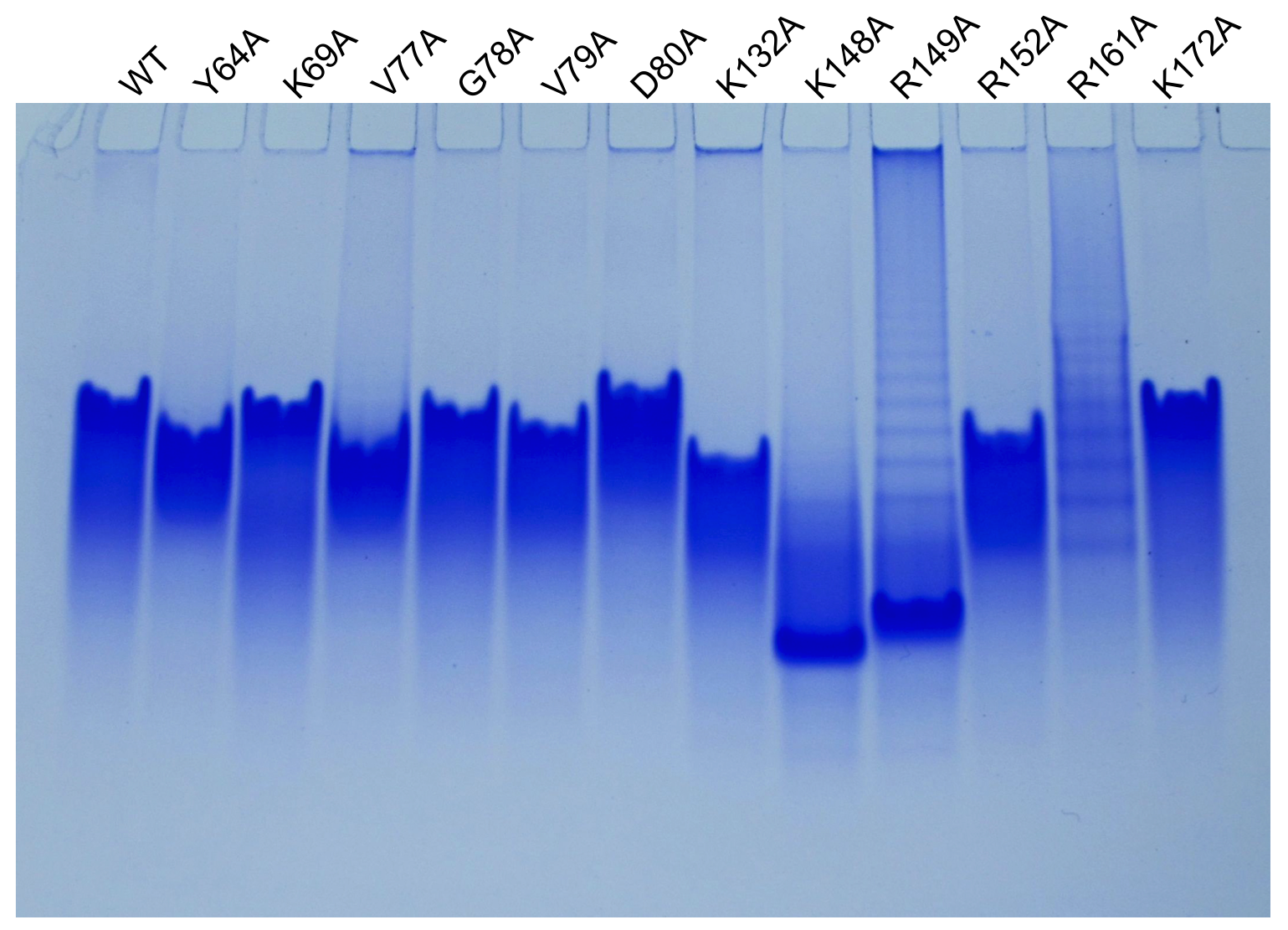

Supplement: Figure S3 — Native PAGE analysis using overloaded samples. Redβ WT and mutant proteins (20 µg per lane) were loaded and run according to the method in Figure 6A. Ladder-like migration patterns were observed for mutants K148A, R149A, and R161A. (TIF) [file pone.0078869.s003.tif]

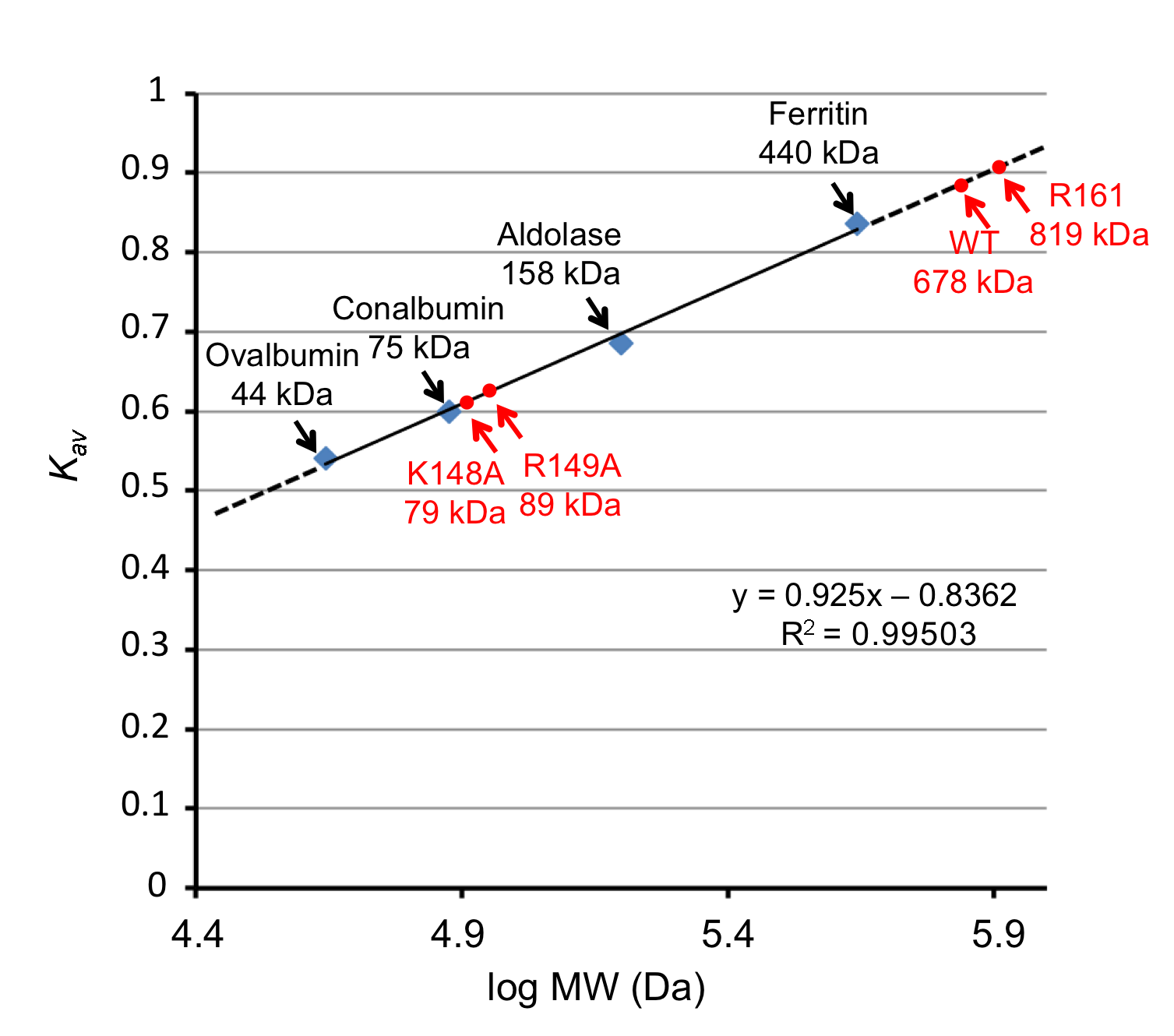

Supplement: Figure S4 — Standard curve for estimation of molecular weights using gel filtration chromatography. Kav values [(Ve - V0)/(Vc - V0)] against log MW of the known protein standards. The generated linear equation and the R2 values are also indicated. The dotted line indicates extrapolation of the standard curve used to estimate the molecular weights of the larger eluting species, such as Redβ WT. (TIF) [file pone.0078869.s004.tif]
