# Supplementary material for: Structural and Functional Characterization of the Redβ Recombinase from Bacteriophage λ
Source: PLoS One. 2013 Nov 11;8(11):e78869. doi: 10.1371/journal.pone.0078869 (PMC3823998; doi:10.1371/journal.pone.0078869)
Supplement: Table S1 — Summary of Redβ mutant analysis and the role of equivalent residues in hRad52. (DOCX) [file pone.0078869.s005.docx]

**Table S1. Summary of Redβ mutant analysis and the role of equivalent residues in hRad52.**

| **Red**β** residue** | **hRad52 residue** | **Conserved in Red**β**?** | **Conserved in both Redβ/Rad52?** | **ssDNA binding (%)** | **2^nd^ DNA strand binding** | **50% Annealing time (min)** | **Quaternary structure** | **TEM ssDNA complex** | **Role in Rad52** |
| --- | --- | --- | --- | --- | --- | --- | --- | --- | --- |
| **Y64** | Y65 | yes | ? (Y) | 98 | ++ | 0.7 | like WT | like WT | ssDNA and dsDNA binding [1,2] |
| **K69** | R70 | yes | ? (K/R) | 94 | + | 15 | like WT | like WT | ssDNA binding [2] |
| **V77** | F79 | yes | hydrophobic (V/F) | 11 | – | 4.3 | like WT | none | ssDNA binding [2] |
| **G78** | G80 | yes | yes | 33 | – | 6.4 | like WT | none | ssDNA binding [2] |
| **V79** | Y81 | yes | hydrophobic (V/Y/F) | 21 | – | 4.5 | like WT | none | ssDNA binding [2] |
| **D80** | N82 | yes | polar (D/N) | 87 | ++ | 0.7 | like WT | weak | ssDNA binding [2] |
| **W82** | W84 | yes | yes | – | – | – | – | – | ssDNA binding [2] |
| **K132** | K133 | no | no | 82 | + | 5.2 | like WT | weak | ss- and dsDNA binding, ternary complex and D-loop formation,,positive supercoiling [3] |
| **K148** | K152 | yes | yes | 43 | – | 2.6 | disrupted | weak | ssDNA binding [1,2] |
| **R149** | R153 | yes | yes | 44 | – | 2.3 | disrupted | none | ssDNA and dsDNA binding [1,2] |
| **R152** | R156 | yes | yes | 13 | – | 3.6 | like WT | none | ssDNA and dsDNA binding [1,2] |
| **R161** |  | yes | no | 11 | – | 4.9 | aggregated | none | – |
| **K172** | K169 | no | only in Rad52 | 76 | + | 2.9 | like WT | weak | ssDNA binding [1], dsDNA binding, D-loop formation (moderate) [3] |

Redβ residues are listed in the first column, while potential equivalents of these in hRad52 (based on sequence alignment) are shown in the second column.

The Redβ W82A mutant was not amenable to purification. For ssDNA binding, the percent binding relative to WT at 3 µM is shown.

**References for Table S1**

1. Kagawa W, Kurumizaka H, Ishitani R, Fukai S, Nureki O, et al. (2002) Crystal structure of the homologous-pairing domain from the human Rad52 recombinase in the undecameric form. Mol Cell 10: 359-371.

2. Lloyd JA, McGrew DA, Knight KL (2005) Identification of residues important for DNA binding in the full-length human Rad52 protein. J Mol Biol 345: 239-249.

3. Kagawa W, Kagawa A, Saito K, Ikawa S, Shibata T, et al. (2008) Identification of a second DNA binding site in the human Rad52 protein. J Biol Chem 283: 24264-24273.
